# Biodegradation of phenol-contaminated soil and plant growth promotion by *Myroides xuanwuensis* H13

**DOI:** 10.1128/spectrum.00266-24

**Published:** 2024-06-25

**Authors:** Zhonghua Wang, Huihong Zhang, Dengxiao Zhang, Yi Wang, Yanlai Han, Xia Xue, Ying Jiang

**Affiliations:** 1 College of Resources and Environment, Henan Agricultural University, Zhengzhou, China; 2 Key Laboratory of Arable Land Conservation (Middle and Lower Reaches of Yangtze River) of the Ministry of Agriculture, College of Resources and Environment, Huazhong Agricultural University, Wuhan, China; 3 Henan Key Laboratory of Helicobacter pylori & Microbiota and Gastrointestinal Cancer, Marshall Medical Research Center, The Fifth Affiliated Hospital of Zhengzhou University, Zhengzhou, China; Instituto de Ecología, A.C. (INECOL), Pátzcuaro, Michoacán, Mexico

**Keywords:** phenol, phenol-degrading bacteria, growth promote, Chinese cabbage, physiological and biochemical interactions

## Abstract

Physicochemical methods for remediating phenol-contaminated soils are costly and inefficient, making biodegradation an environmentally friendly alternative approach. This study aims to screen for potential phenol-degrading bacteria and to verify the removal capacities of a selected strain in a bioaugmentation experiment at the greenhouse level using *Brassica chinensis* L. (Chinese cabbage) as the model plant and phenol-contaminated soil. In parallel, pot experiments were conducted using a collaborative approach based on this model system. We found that *Myroides xuanwuensis* strain H13 showed a high degradation capability, with a 97.67% efficiency in degrading 100 mg/L phenol. Under shaking flask conditions, H13 facilitated the solubilization of tricalcium phosphate and potassium feldspar powder. Pot experiments suggested a phenol removal percentage of 89.22% and enhanced availability of soil phosphorus and potassium for plants with H13 inoculation. In this case, the abundance of soil microbes and the activity of soil enzymes significantly increased as well. Furthermore, both photosynthesis and the antioxidant system in Chinese cabbage were enhanced following H13 inoculation, resulting in its increased yield and quality. Partial least squares path modeling revealed that H13 can primarily affect plant root growth, with a secondary impact on photosynthesis. These findings highlight the potential of biodegradation from phenol-degrading bacteria as a promising strategy for efficient phenol removal from soil while promoting plant growth and health.

**IMPORTANCE:**

This study is significant for environmental remediation and agriculture by its exploration of a more environmentally friendly and cost-effective bio-strategy in treating phenol-contaminated soil. These findings have essential implications for environmental remediation efforts and sustainable agriculture. By utilizing the biodegradation capabilities of *Myroides xuanwuensis* strain H13, it is possible to remove phenol contaminants from the soil efficiently, reducing their negative effects. Furthermore, the enhanced growth and health of the Chinese cabbage plants indicate the potential of this approach to promote sustainable crop production.

## INTRODUCTION

Phenol is a crucial organic chemical and is commonly used in the production of resins, fungicides, preservatives, and pharmaceuticals (1). It is essential for manufacturing synthetic fibers, rubbers, and dyes (2, 3). As a toxic organic compound, the mass production of phenol and its derivatives brings the inevitable harm to the natural ecosystem and human health. Phenol relics can affect the soil’s organic matter, microorganisms, and enzyme activity, leading to soil permeability reduction, moisture and nutrient loss, and fertility decline. In parallel, phenol enters the crops through root absorption and foliar deposition, disturbing the growth and development of plants and reducing the crop yield and quality. Notably, phenol can be transmitted through the food chain to enter humans and livestock, causing damage to the nervous system, liver, kidney, and other organs, which even results in genetic mutations and cancer (4). The mobility of phenol is limited compared with it in soil due to sorbing to soil particles; thus, its removal in terrestrial environments can be challenging (5).

The physical and chemical methods for soil remediation that is contaminated with phenol primarily include thermal treatment, leaching, electrokinetics, and chemical oxidation (6, 7). These methods can effectively reduce phenol concentration in the soil but with obvious drawbacks, such as high cost, limited applicability, and significant impacts on soil nutrients and fertility (8). Microbial remediation strategy uses indigenous or exogenous induced microbial populations within the contaminated soil to reduce the content of soil organic pollutants (9, 10). Specifically, phenol-degrading microorganisms utilize oxygen to break down organic contaminants into smaller and less toxic compounds (11). Some anaerobic bacteria and archaea use alternative electron acceptors, for instance, nitrate, sulfate, or carbon dioxide, to degrade the organic pollutants, causing the production of simpler end products, including methane or carbon dioxide (12). Phenol-degrading bacteria can be isolated from soil, water, and sediments, and they possess the ability to utilize phenol as a carbon source, breaking it down into less-toxic particles under a wide range of environmental conditions. Up to date, a series of phenol-degrading bacteria have been confirmed to be effective for bioremediation, such as *Pseudomonas*, *Achromobacter*, *Rhodococcus*, *Acinetobacter*, *Bacillus*, and *Ralstonia eutropha* (13–16).

Recent studies indicate that plants possess the capacity to tolerate and eliminate environmental pollutants by phytoremediation (17, 18). However, the effectiveness of phytoremediation for volatile organic pollutants (e.g., toluene and phenol) is compromised due to the limited capacity of plants to degrade such pollutants and the potential phytotoxicity or volatilization of these chemicals through leaves (19). The symbiotic relationship between plants and microorganisms has been observed to accelerate the degradation of organic pollutants in soil (20, 21). This phenomenon is facilitated by the exchange of root exudates and decomposition products, which provide nutrients to microorganisms, as microbial activity promotes the release of root exudates (10, 22). Despite of these insights, limited research investigated the impact of soil antioxidant enzyme activity and plant responses during the degradation of soil-bound phenol under the combined influence of microorganisms and plants.

A key challenge in phytoremediation is the tendency of high concentrations of pollutants to hinder plant growth, often through oxidative stress, which subsequently decreases the rate of in-situ phytoremediation (23). This process generates a significant amount of reactive oxygen species (ROS) to interact with the nutrient cycling, including superoxide (O_2_•−), hydroxyl radicals (OH∙), hydrogen peroxide (H_2_O_2_), and singlet oxygen (24, 25). In response, plants have evolved diverse protective and repair mechanisms to mitigate oxidative damage, employing both enzymatic and non-enzymatic antioxidant systems (26–30). The array of enzymatic antioxidants comprises peroxidase (POD), superoxide dismutase (SOD), ascorbate peroxidase (APX), catalase (CAT), and glutathione reductase, while the well-known non-enzymatic antioxidants include glutathione, ascorbate, carotenoids, and tocopherols (31). Therefore, it is important to study how plants and microorganisms together affect soil antioxidant enzymes and the plant’s antioxidant systems when dealing with soil organic pollutants. This study aims to screen phenol-removal bacteria with growth-promoting capabilities in a vegetable field that has been exposed to prolonged phenol contamination. Pot experiments were conducted to investigate the remediation of phenol-contaminated soil using a collaborative approach involving phenol-degrading bacteria and Chinese cabbage. Furthermore, we assessed the influence of phenol-degrading bacteria on soil antioxidant enzymes and the plant’s antioxidant system.

## MATERIALS AND METHODS

### Soil sample

The soil used in this study was collected from Guanduqiao village, Zhongmou City, Henan Province (34.7480°N, 114.0452°E). The area is in the mid-latitude warm temperate continental monsoon climate. Its annual average sunshine is 2,366 h, the annual average temperature is 14.2°C, and the annual average precipitation is 616 mm. The average agricultural period in the region is 309 days/year, while vegetables and fruits are mainly grown in greenhouses.

The soil samples were collected from the greenhouse at a depth of 0–20 cm that have been subjected to pesticide pollution (carbofuran, triadimefon, and difenoconazole) for over 10 years. The soil type was fluvo-aquic with a light texture, and the basic physical and chemical properties of the soil were measured: pH 7.21, organic matter 10.27 g/kg, total nitrogen 1.07 g/kg, alkali hydrolyzable nitrogen 74.20 mg/kg, available phosphorus 30.68 mg/kg, available potassium 116.75 mg/kg, and phenol 56.26 mg/kg.

### Isolation of phenol-degrading bacteria from phenol-contaminated soil

Ten gram of contaminated soil was blended with sterile water and subjected to agitation at 30°C with a rotational speed of 180 rpm for 30 minutes. The mixture was allowed to settle, and the supernatant was drawn off. Subsequently, the supernatant was appropriately diluted before being spread onto a solid Luria-Bertani (LB) medium. The culture plates were then placed in an incubator at 30°C for 24 h, adhering to the methodology established by A. G. Wollum (32).

Colonies displayed diverse morphologies, and colors were systematically assigned identification numbers. All selected colonies were adequately maintained at 4°C for the subsequent evaluation of their phenol removal efficacy.

The isolated and purified strains were inoculated in LB liquid medium and cultured in shaking plates at 30°C 180 r/min for 2 days, then centrifuged at 4,000 r/min for 15 min. The supernatant was discarded and, the bacterial pellet was collected and washed with sterilized water 2–3 times. The cell concentration was adjusted to make it OD_600_ = 1 and stored as seed solution at 4°C.

The 1 mL of bacterial suspension was inoculated in a 50 mL basic medium with phenol as the sole carbon source. The basic medium contained: FeSO_4_·7H_2_O (0.018 g), K_2_HPO_4_·3H_2_O (1.31 g), MgSO_4_·7H_2_O (0.50 g), NaNO_3_ (3.0 g), and KCl (0.50 g), in 1,000 mL double distilled water (33). The phenol concentration was increased gradually using 10 mg/L, 50 mg/L, and 100 mg/L. This set was based on the prior study by combining the phenol pollution status of the sampled soil (34). The concentration of the bacterial suspension was adjusted to an OD_600_ = 1. Then, the strain was inoculated in the basic medium with 100 mg/L phenol as the sole carbon source. The medium without bacterial suspension inoculation was set as the control (three replications). All mediums were incubated at 28°C with 180 r/min for 24 h. After shaking, the content of phenol in each culture bottle was determined by the modified 4-amino antipyrine method (35, 36). The medium was centrifuged at 12,000 r/min for 1 min, and then 30 µL of supernatant was added into a 10 mL test tube. Sequentially, we added 4 mL of distilled water, 40 µL of ammonia buffer solution (pH 10), 80 µL of 2% 4-aminoantipyrine, and 80 µL of 80% potassium ferricyanide solution to the test tube and mixed well. The tube stayed still for 15 min, and its absorbance value was measured at 510 nm. The absorbance data were converted to phenol concentrations using a calibration curve from 0 to 100 mg/L. The strain with the highest removal percentage was selected as the phenol-degrading bacteria in the present work.

Removal percentage = (phenol content of control culture solution − phenol content of bacterial solution)/phenol content of control culture solution × 100%.

### The phosphorus-solubilizing and potassium-dissolving ability

The 50 mL of PKO (Pikovaskaia’s inorganic phosphorus) medium was added into a 250 mL conical flask and inoculated with 1 mL of phenol-degrading bacteria at an OD_600_ of 1 (three replications) (37). The flask was incubated for 72 h at 30°C with a rotational speed of 180 r/min. Then, we collected the culture broth and centrifuged it at 4°C for 10 min at 10,000 r/min. The concentration of phosphorus was determined by the molybdenum antimony colorimetric assay method (38).

The 50 mL of potassium-solubilizing bacteria culture medium was added and inoculated with 1 mL of phenol-degrading bacteria at an OD_600_ of 1 into a 250 mL triangular flask (three replications) (39). The flask was incubated for 72 h at 30°C with a rotational speed of 180 r/min. Then, we collected the culture broth and centrifuged it at 4°C for 20 min at 6,000 r/min. The supernatant was collected, and the potassium content was measured using the flame photometry method (40).

### Morphological, physiological, biochemical, and molecular characterization

The selected phenol-degrading bacteria were inoculated on LB agar plates and incubated at 30°C for 24 h. The morphology of colonies, including size, shape, color, shine, texture, and transparency, was observed using a microscope (SK200, Motic). The Gram-staining method was employed for strain identification (41). The selected phenol-degrading bacteria were inoculated in an LB liquid medium to prepare a bacterial suspension. The bacterial cells were washed with phosphate buffer and then fixed with 2.5% glutaraldehyde. After another wash with phosphate buffer, gradient dehydration was performed using ethanol-water solutions (42). The morphology and size of the bacterial samples were observed by scanning electron microscope (SEM; S-3400N, Hitachi) at the Central Laboratory of Henan Agricultural University. Aerobic test, contact enzyme determination, starch hydrolysis test, methyl red test, phthalein methyl methanol test (VP test), gelatin hydrolysis test, and citrate utilization test were conducted in the present study by the identification manual of common bacteria (41).

The selected phenol-degrading bacteria were characterized through 16S rRNA sequence alignment. The 16S rRNA sequence amplification was carried out using PCR and employed universal primers: 27F (5′-AGAGTTTGATCCTGGCTCAG-3′) and 1492R (5′-GGTTACCTTGTTACGACTT-3′). The amplified samples were sequenced by Sangon Biotech Co., Ltd. (Shanghai, China), according to the method outlined by Monis et al. (43). The acquired sequences underwent a BLAST analysis against the NCBI (National Center for Biotechnology Information) database, and a 16S rDNA phylogenetic tree was constructed using the neighbor-joining method with a bootstrap value of 1,000 by employing MEGA 7.0 software, as described by Kumar et al. (44). All sequences were deposited in the GenBank repository, and the corresponding accession numbers were obtained.

### Growth and phenol degradation of phenol-degrading bacteria under different culture conditions

The selected phenol-degrading bacteria was tested in media with different pH values (3, 4, 5, 6, 7, 8, and 9), inoculation times (12, 24, 48, 72, and 120 h), initial phenol concentrations (100, 250, 500, 1,000, 1,500, and 2,000 mg/L), inoculation amounts (1%, 5%, 10%, 15%, and 20%), liquid volumes (25, 50, 75, 100, 150, and 250 mL), and nitrogen source (peptone, yeast extract, sodium nitrate, urea, and ammonium sulfate) to evaluate the effect of potential phenol-degrading bacteria on bacteria growth (OD_600_) and phenol degradation.

### Effect of phenol-degrading bacteria on plant growth in greenhouse

The Chinese cabbage (*Brassica chinensis* L.) variety “April slow” was sampled in this experiment. The soil used in this experiment was collected from the field containing isolated phenol-degrading bacteria. The impurities, such as roots, stones, and soil animals, were removed and sifted through 10 mesh sieve (1.70 mm), then mixed well. The amount of soil in each basin was 1 kg. The culture broth of the collected bacteria was centrifuged to remove the supernatant. The bacterial pellet of centrifugation and resuspension was repeated three times using sterile water, and the concentration was adjusted to 10^11^ CFU(colony-forming unit)/mL.

The pot experiment was set up with six treatments: (i) phenol-contaminated soils (P); (ii) phenol-contaminated soils + inactivated bacteria (PI); (iii) phenol-contaminated soils + active bacteria (PB); (iv) phenol-contaminated soils + vegetable (PV); (v) phenol-contaminated soils + inactivated bacteria + vegetable (PIV); (vi) phenol-contaminated soils + active bacteria + vegetable (PBV).

The Chinese cabbage seeds were sterilized with H_2_O_2_ and germinated on sterilized filter paper sheets in petri dishes for 3 days. Six uniform seedlings were then transplanted into each pot and transferred to a greenhouse. The greenhouse was set up as a light/dark period of 16/8 h, a relative humidity of 60% ± 5%, and a temperature of 25°C. After 1 week of emergence, three plants with uniform growth were retained in each pot. The inoculum of phenol-degrading bacteria was 10^8^ CFU/g soil. Treatment of inactivated strains was inoculated with high temperature and high pressure inactivated strains. Each treatment was set up with four replicates. The potted experiment was carried out in the greenhouse and watered regularly every day to keep the soil water content at 60%–70% of the field capacity. Chinese cabbage was sampled after 35 days of strain inoculation.

### Measurement of soil phenol, nutrients, microbial biomass, and enzyme activity

Soil samples were collected for each treatment sifted through 10 mesh sieves and mixed well. Soil phenol content was determined by Gas chromatography-mass spectrometry (11). The content of available phosphorus and potassium in soil samples was measured after air-drying (45). The microbial biomass carbon (MBC) and microbial biomass nitrogen (MBN) were determined by a chloroform fumigation-direct extraction method (46, 47). Soil base respiration (SBR) was measured by a Gas chromatography system (GC-2014, Shimadzu, Kyoto, Japan) based on the linear increase in gas with time (48). The microbial metabolic quotient (qCO_2_ = SBR/MBC) was calculated with the formula of T. Anderson and K. Domsch (49). Soil dehydrogenase activity (S-DHA) was determined by taking 1 g of soil and incubating it with triphenyl tetrazolium chloride for 6 h at 30°C (50). The determination of soil catalase activity (S-CAT) was conducted using the UV spectrophotometric method (51). The soil polyphenol oxidase activity (S-PPO) was determined using the pyrogallol colorimetric method (52). The determination of soil urease activity (S-UE) was conducted using the Nesslerization colorimetric method (53).

### Measurement of photosynthetic system, biomass, and root system architecture in Chinese cabbage

The photosynthetic rate (Pn), stomatal conductance (Gs), intercellular CO_2_ concentration (Ci), and transpiration rate (Tr) of the first fully expanded leaf on the top of the crown were measured by a portable photosynthesis system (Li-6400, LICOR Inc., USA) before harvest. During the measurement, the relative humidity and air temperature in the greenhouse were kept at 60% ± 5% and 25°C. Leaf temperature was maintained at 25°C, and photosynthetic photon flux density in the leaf cuvette was set at 1,000 µmol/m^2^/s. Three leaves were randomly measured from each treatment.

The Chinese cabbage plants were washed with tap water to remove the soil from the roots and then stored in 70% alcohol. The main plant height, leaf length, leaf width, and development (maximum width of the plant on a horizontal projection plane) were measured with a ruler (scale: 1 mm). The fresh and dry weights (all samples were dried at 105°C for 30 min and then at 70°C until a constant weight) were weighed with balance (scale: 0.01 g). The root images were taken by using a scanner (LA1600+ scanner, Canada). The root system was divided into five categories based on root diameter (RD): I (RD 0–0.5 mm), II (RD 0.5–1.0 mm), III (RD 1.0–1.5 mm), IV (RD 1.5–2.0 mm), and V (RD > 2.0 mm). The root-related parameters, including root length, root surface area, root volume, RD, and root tips, were analyzed using WinRhizo software (WinRhizo2003b, Canada).

The first fully expanded leaves on the top of the crown, including the leaf used for gas exchange measurement, were cut into small pieces, and 0.3 g samples were soaked in 25 mL 95% ethanol. Samples were placed in a dark environment at room temperature. After the color of the leaves had faded, the content of photosynthetic pigments, including chlorophyll a (Chl a) and b (Chl b), and carotenoid was measured at 470 nm, 649 nm, and 665 nm by a UV–Visible spectrophotometer. The total chlorophyll (Total Chl) content is equal to the sum of Chl a and Chl b content (54).

### Measurement of quality, resistance substances, and enzyme activity in Chinese cabbage

The collected fresh Chinese cabbage samples were separated into the aboveground and underground parts. The quantification of reducing sugars was performed using the 2,4-dinitrosalicylic acid colorimetric method (55). Vitamin C (VC) content was quantified using the indophenol method (56). The determination of crude fiber content was conducted using the gravimetric method (57). The determination of nitrate content was carried out following Singh’s method (58). Malonaldehyde (MDA) content was measured by the thiobarbituric acid method reaction (59). The concentration of H_2_O_2_ in the leaves was determined using Alexieva’s method (60). SOD was determined by the nitroblue tetrazolium method based on Tandy’s studies (61). CAT was assayed according to H. Aebi (62). POD was determined according to Zhang et al. (63). APX was measured based on the method of Y. Nakano and K. Asada (64).

### Statistics

Statistical analyses were carried out using SPSS 16.0 (SPSS Inc., Chicago, IL, USA). One-way ANOVA (Analysis of Variance) with a least significant difference test (*P* < 0.05) was used to determine the significance of differences (65, 66). A two-way ANOVA was employed to assess the effects of bacteria, plants, and their interaction. Pearson’s correlation analysis was performed to investigate the relationships between different indicators. Before performing PCA (principal component analysis) in Metabo Analyst 5.0, all data were log-transformed. ClustVis was utilized to create PCA plots and heatmaps (67). All graphs were generated using Origin 2018 (OriginLab Corporation, Northampton, MA, USA). We explored the relationship between soil microbial activity, soil enzyme activities, plant root system, plant antioxidant system, and plant biomass and quality by using partial least squares path modeling (PLS-PM), a particularly useful statistical method for demonstrating cause and effect relationships among observed and latent variables (68). The estimates of path coefficients and the coefficients of determination (*R*^2^) in our path model were validated by R (v. 3.3.3) with the package “plspm” (1,000 bootstraps).

## RESULTS

### Screening of phenol-degrading bacteria

**Fig 1:**
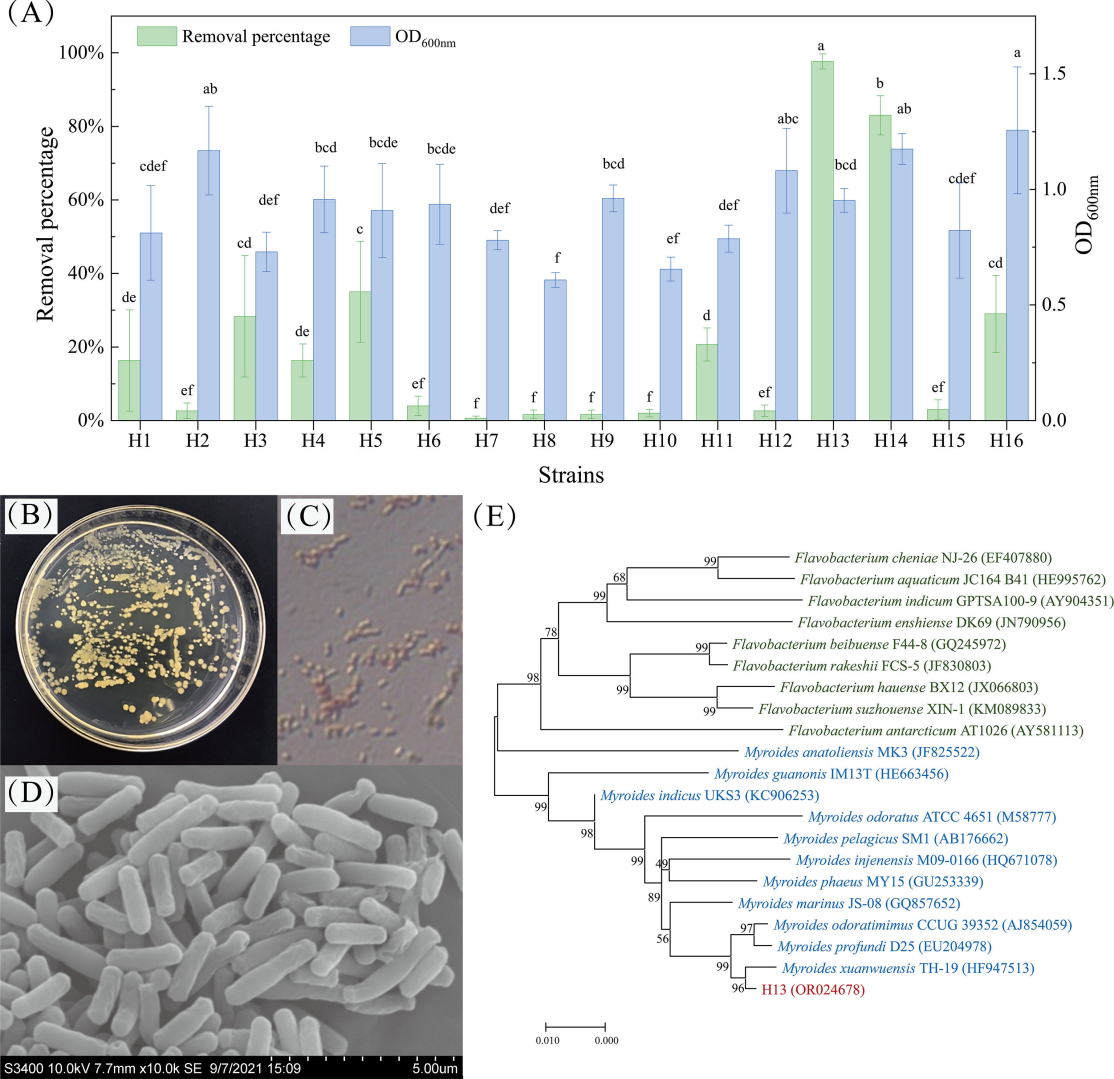
The phenol removal percentage and growth status of different strains after 24 h with 100 mg/L of phenol (**A**). Identification of the strain H13. The colony morphology of H13 (**B**). Gram staining of H13 (**C**). SEM image of H13 at 10,000× (**D**). Phylogenetic tree of H13 established with 16S rRNA sequences (**E**). Note: H1–H16 represents the numbering of different strains. Different letters show significant differences (*P* < 0.05).

A total of 16 strains potentially capable of degrading phenol were screened and isolated. After 48 h of cultivation in an inorganic salt medium with a single phenol as a carbon source, strain H13 exhibited the highest removal capability toward 100 mg/L phenol, achieving a remarkable degradation efficiency of 97.67%. Simultaneously, the growth of strain H13 was measured to be 0.94 (OD_600_; Fig. 1A). After 72 h, strain H13 was able to dissolve tricalcium phosphorus and potassium feldspar to concentrations of 325.62 mg/L and 25.99 mg/L, respectively.

The surface of strain H13 was smooth with a neat, opaque, and slightly yellow edge (Fig. 1B). Gram staining showed that strain H13 was Gram-negative and irregular rod (Fig. 1C). SEM observation found that the H13 was rod-shaped form with a size of 0.56–0.57 µm × 1.3–2.94 µm (Fig. 1D). H13 showed negative for gelatin hydrolysis, V-P test, methyl red test, citrate utilization test, and hydrogen sulfide test. However, it showed positive in the contact enzyme test and starch hydrolysis test (Table 1).

**TABLE 1:** Physiological and biochemical characteristics of strain H13^*a*^^,^^*b*^

Indicators	Results	Indicators	Results
Gram stain	Gram-negative	Starch hydrolysis	+
Aerobic test	Aerobic	Gelatin liquefaction	−
Contact enzyme test	+	Sulfur-containing amino acid	−
Methyl red reaction	−	Citrate utilization	−
V-P test	−		

^*a*^ Note: +: positive reaction; −: negative reaction.

^*b*^ V-P test: Voges-Proskauer test.

The partial sequence of the 16S rRNA gene was a continuous stretch of 1,329 bp (OR024678). Based on the neighbor-joining methods, a phylogenetic tree was constructed and indicated that the most homologous strain of H13 was *Myroides xuanwuensis* TH-19 (Fig. 1E). Therefore, the strain H13 was identified and affiliated with *M. xuanwuensis*.

### The growth and phenol degradation of strain H13 under different conditions

**Fig 2:**
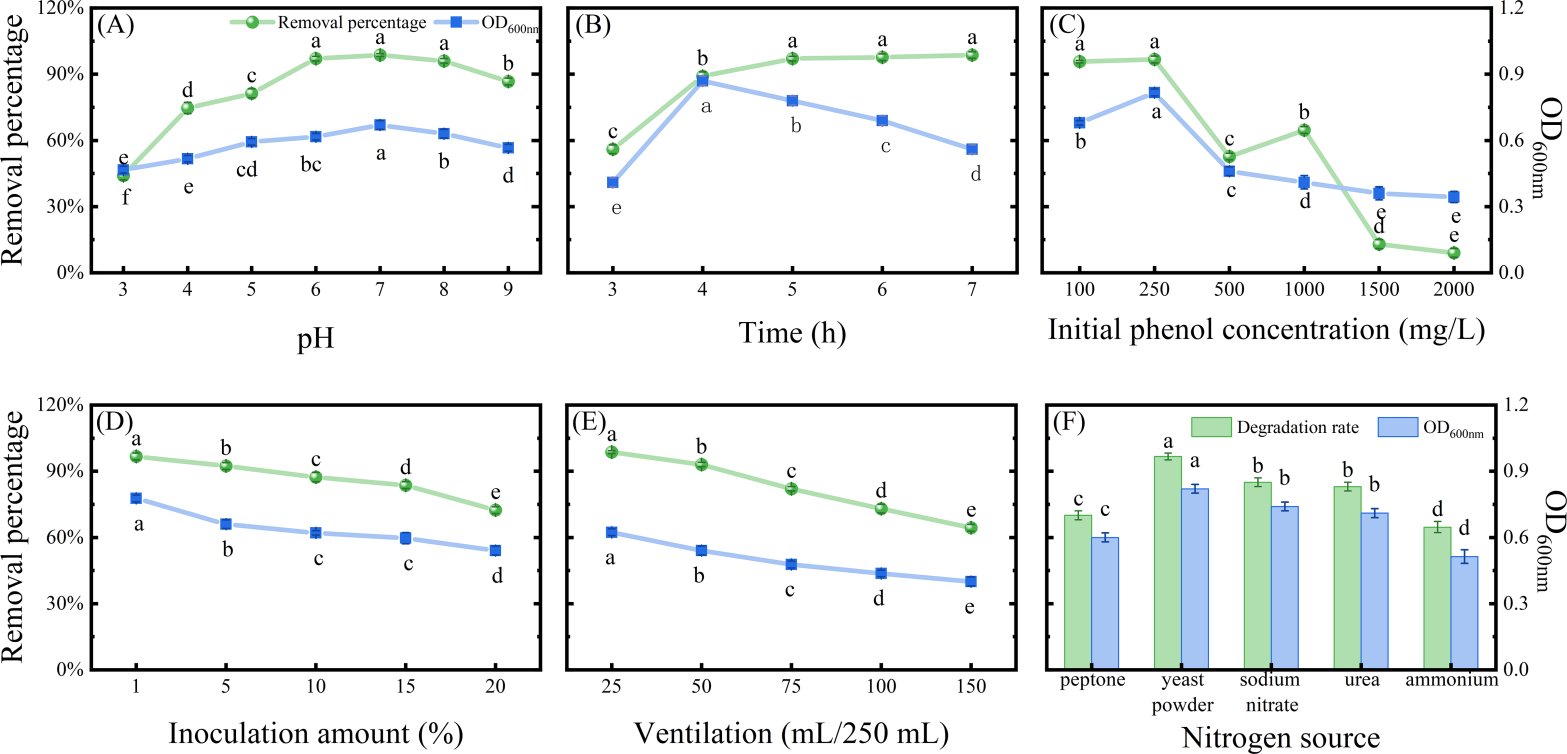
The effects of different initial conditions on the phenol degradation ability and growth of bacterial strains. Different pH conditions (**A**). Different time points (**B**). Different initial concentrations of phenol (**C**). Different inoculation levels (**D**). Different volumes of liquid loading (**E**). Different nitrogen sources (**F**). Different letters show significant differences (*P* < 0.05).

The highest removal percentage of phenol degradation, up to 98.67%, was shown by strain H13 at pH 7. The phenol degradation and OD600 value gradually increased when the pH was below 7 but decreased when the pH was above 7 (Fig. 2A). Within 24 h, strain H13 exhibited rapid growth, with OD600 reaching its maximum, and the removal percentage reaching 97.00% at 48 h. Afterward, the removal percentage remained relatively stable. The culture medium’s nutrients were consumed over time, reducing bacterial growth (Fig. 2B).

There was no significant difference (*P* < 0.05) in the phenol removal percentage between the initial concentrations of 100 mg/L and 250 mg/L. As the initial concentration of phenol increased, the removal percentage gradually decreased. When phenol reached 2,000 mg/L, degradation dropped to 9.00%. After the initial concentration of phenol exceeded 250 mg/L, the growth of the strain started to decline. Therefore, the optimal degradation concentration for strain H13 was between 100 mg/L and 250 mg/L (Fig. 2C).

Under the inoculation amount was 1% condition, strain H13 showed the highest removal percentage of phenol, reaching up to 96.67%. However, as the inoculation amount increased, the removal percentage decreased, although it remained above 70%. The growth of bacteria, as indicated by the OD600 value, showed a significant decrease (*P* < 0.05) with the increase of inoculation amount (Fig. 2D).

Compared to other treatments, a 25 mL bacterial suspension in the triangular flask indicated significantly higher phenol degradation (*P* < 0.05). As the liquid volume increased and the oxygen volume in the flask decreased, the phenol degradation ability of strain H13 showed a downward trend. Simultaneously, the OD_600_ value also demonstrated a significant decrease with the increase in liquid volume (*P* < 0.05; Fig. 2E).

Different nitrogen sources showed varying effects on the phenol degradation ability of strain H13. When yeast powder was used as the nitrogen source, strain H13 had the highest phenol removal percentage and the best growth. Under ammonium sulfate was used as the nitrogen source condition, the phenol degradation ability of strain H13 was the lowest. However, it is worth noting that the removal percentage of all nitrogen sources was still above 60% (Fig. 2F).

### The effect of strain H13 on phenol and nutrient content in potted soil

**Fig 3:**
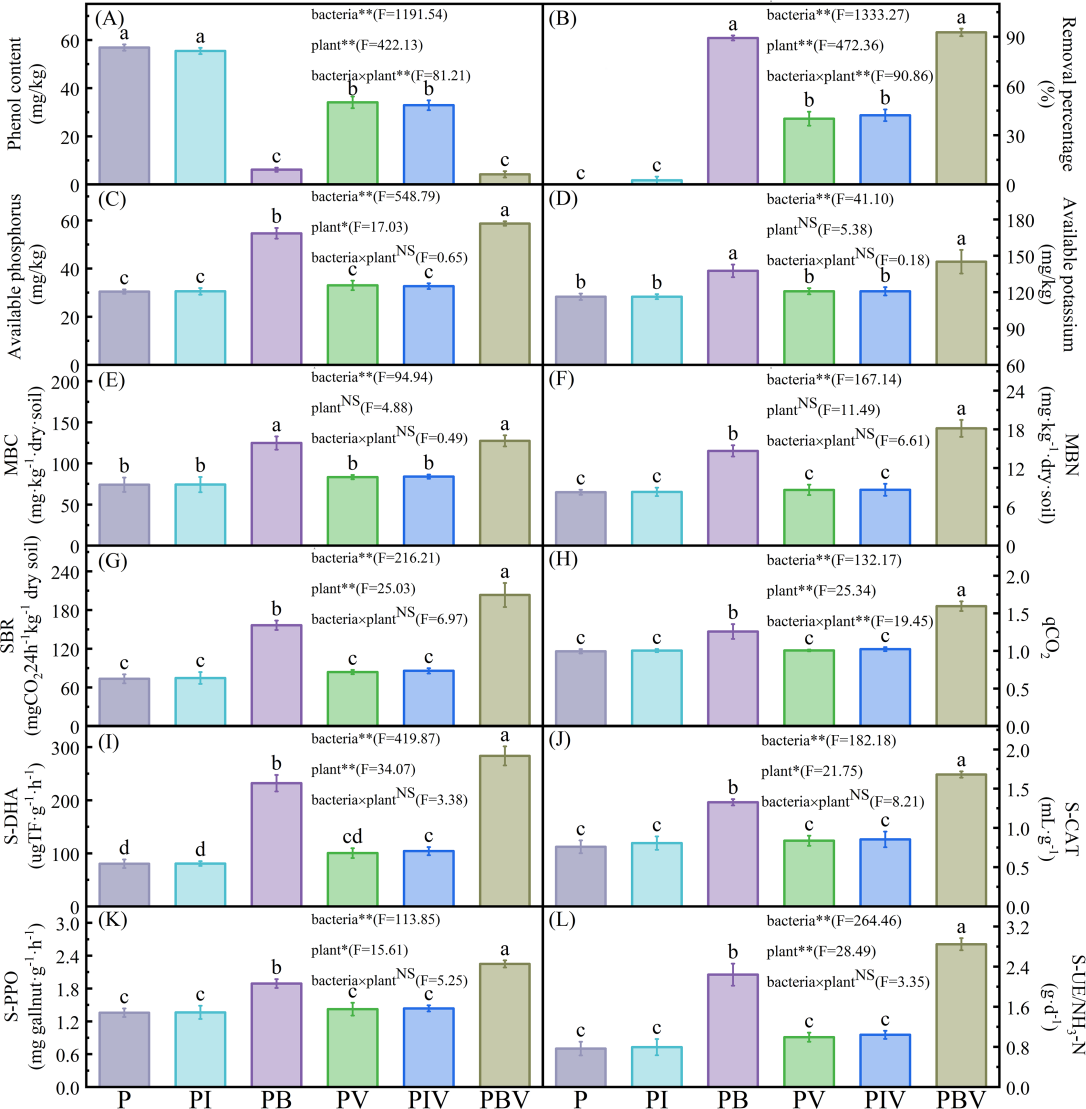
The effects of inoculating phenol-degrading bacteria and planting Chinese cabbage on soil phenol content (**A**), removal percentage of phenol (**B**), soil available phosphorus content (**C**), soil available potassium content (**D**), SMC (**E**), SMN (**F**), SBR (**G**), qCO_2_ (**H**), S-DHA (**I**), S-CAT (**J**), S-PPO (**K**), and S-UE (**L**). MBC: Microbial biomass carbon; MBN: Microbial biomass nitrogen. P = phenol-contaminated soils; PI = phenol-contaminated soils + inactivated bacteria; PB = phenol-contaminated soils + active bacteria; PV = phenol-contaminated soils + vegetable; PIV = phenol-contaminated soils + inactivated bacteria + vegetable; PBV = phenol-contaminated soils + active bacteria + vegetable. Different letters show significant differences (*P* < 0.05). Two-way ANOVA was used to show the effect of bacterium, plants, and their interaction. F-value indicates a significance level based on the results of two-way ANOVA. NS showed non-significance at the 95% level (*, *P* < 0.05; **, *P* < 0.01).

The interaction between the inoculated strain and the cultivation of Chinese cabbage showed an impact on the soil’s phenol content (significant bacteria × plant). The soil phenol content in PB and PBV treatments decreased significantly by 89.22% and 92.63% (*P* < 0.05), respectively. However, PV and PIV treatments only decreased by 40.05% and 42.12%, respectively (Fig. 3A and B). Compared with the phosphorus treatment, the soil-available phosphorus and potassium in PB treatments significantly increased by 79.47% and 18.40% (*P* < 0.05) and in PBV treatments significantly increased by 92.81% and 24.90% (*P* < 0.05; Fig. 3C and D). While PI, PV, and PIV treatments did not increase significantly. Therefore, inoculating soil with the active strain H13, along with the combined action of H13 and native microorganisms, can notably decrease the phenol levels and significantly boost the nutrient levels in the soil.

### The effect of strain H13 on soil microbial biomass and soil enzyme activity

The PB and PBV treatments with active inoculants had higher MBC and MBN levels than the others (Fig. 3E and F). A similar trend was observed in SBR and qCO_2_ (Fig. 3G and H). There were significant variations in the activities of S-DHA, S-CAT, S-PPO, and S-UE among the six treatments (*P* < 0.05). The PB treatment displayed significantly higher activity compared to the P, PI, PV, and PIV treatments, while the PBV treatment exhibited significantly higher activity than all the other treatments (*P* < 0.05; Fig. 3I through L).

### The effect of strain H13 on the growth of Chinese cabbage

No significant differences were observed in the growth of Chinese cabbage between the PIV and PV treatments, in which the strains were inoculated with inactivated bacteria. A significant difference (*P* < 0.05) was found between the growth parameters from treatments PVB and PV. The plant height, leaf length, leaf width, fresh weight, dry weight, and leaf area from PVB treatment showed a 54.82%, 46.08%, 43.43%, 155.21%, 158.33%, and 59.45% increase, respectively (Table 2). The plant root length, root surface area, root average diameter, root tips, and root forks from PVB treatment were 60.61%, 73.26%, 88.20%, 33.62%, and 72.71% increased as well (Table 2). These findings indicate that the PBV treatment primarily influenced the root morphology within the diameter range of 0–1.5 mm. Root length (RD 0–1.5 mm), root surface area (RD 0–1.5 mm), and root volume (RD 0–1.5 mm) increased by 60.01%, 64.33%, and 63.11%, respectively (Table 2).

**TABLE 2:** The effects of inoculated strains on the above-ground growth and the roots of Chinese cabbage at different diameter levels^*a*^

Class	PV	PIV	PBV
Leaf length (cm)	9.76 ± 0.64 b	9.90 ± 0.52 b	15.11 ± 1.11 a
Leaf width (cm)	5.36 ± 0.30 b	5.30 ± 0.43 b	7.83 ± 0.20 a
Fresh weight (g)	3.73 ± 0.23 b	3.63 ± 0.15 b	5.35 ± 0.25 a
Dry weight (g)	3.26 ± 0.17 b	3.41 ± 1.33 b	8.32 ± 0.68 a
Number of blades	0.24 ± 0.09 b	0.26 ± 0.12 b	0.62 ± 0.01 a
Development (cm)	11.00 ± 1.00 a	11.66 ± 1.52 a	11.33 ± 0.57 a
Leaf length (cm)	12.33 ± 0.57 b	14.66 ± 2.51 b	19.66 ± 0.57 a
Total root length	868.23 ± 33.63 b	862.12 ± 32.7 b	1394.45 ± 22.61 a
I (RD 0–0.5 mm)	596.06 ± 67.63 b	567.83 ± 59.64 b	910.13 ± 102.12 a
II (RD 0.5–1.0 mm)	150.13 ± 13.22 b	162.69 ± 14.06 b	269.71 ± 22.2 a
III (RD 1.0–1.5 mm)	57.21 ± 8.37 b	62.79 ± 9.17 b	105.72 ± 16.98 a
IV (RD 1.5–2.0mm)	26.87 ± 7.07 a	27.79 ± 3.33 a	42.15 ± 13.19 a
V (RD >2.0 mm)	36.9 ± 6.3 a	39.87 ± 6.23 a	65.3 ± 27.9 a
Total root surface area	159.29 ± 12.68 b	168.36 ± 11.84 b	275.98 ± 38.4 a
I (RD 0–0.5 mm)	38.32 ± 3.36 b	37.55 ± 2.02 b	60.1 ± 4.69 a
II (RD 0.5–1.0 mm)	31.33 ± 2.77 b	34.03 ± 2.86 b	56.47 ± 4.96 a
III (RD 1.0–1.5 mm)	21.7 ± 3.17 b	23.82 ± 3.49 b	40.12 ± 6.71 a
IV (RD 1.5–2.0 mm)	14.66 ± 3.92 a	15.19 ± 1.86 a	23.05 ± 7.31 a
V (RD >2.0 mm)	36.05 ± 4.69 a	38.28 ± 5.22 a	65.77 ± 28 a
Total root volume	2.34 ± 0.47 b	2.63 ± 0.43 b	4.41 ± 1.34 a
I (RD 0–0.5 mm)	0.23 ± 0.01 b	0.23 ± 0 b	0.38 ± 0.01 a
II (RD 0.5–1.0 mm)	0.54 ± 0.04 b	0.59 ± 0.04 b	0.98 ± 0.09 a
III (RD 1.0–1.5 mm)	0.66 ± 0.09 b	0.73 ± 0.1 b	1.23 ± 0.21 a
IV (RD 1.5–2.0mm)	0.64 ± 0.17 a	0.66 ± 0.08 a	1.01 ± 0.32 a
V (RD >2.0 mm)	3.13 ± 0.27 b	3.27 ± 0.46 ab	6.29 ± 2.56 a
Root tips	2865.33 ± 406.45 ab	2,620 ± 435.54 b	3828.66 ± 657.35 a
Root forks	6598.66 ± 134.76 b	6428.33 ± 329.73 b	11396.66 ± 207.49 a
Root crossings	1,837 ± 215.93 b	1674.33 ± 334.57 b	3273.66 ± 350.82 a

^*a*^ Note: PV = phenol-contaminated soils + vegetable; PIV = phenol-contaminated soils + inactivated bacteria + vegetable; PBV = phenol-contaminated soils + active bacteria + vegetable. Different letters show significant differences (*P* < 0.05). The root system was divided into five categories based on RD: I (RD 0–0.5 mm), II (RD 0.5–1.0 mm), III (RD 1.0–1.5 mm), IV (RD 1.5–2.0 mm), and V (RD >2.0 mm).

### The effect of strain H13 on photosynthesis, quality, resistance substances, and resistance enzyme activity of Chinese cabbage

**Fig 4:**
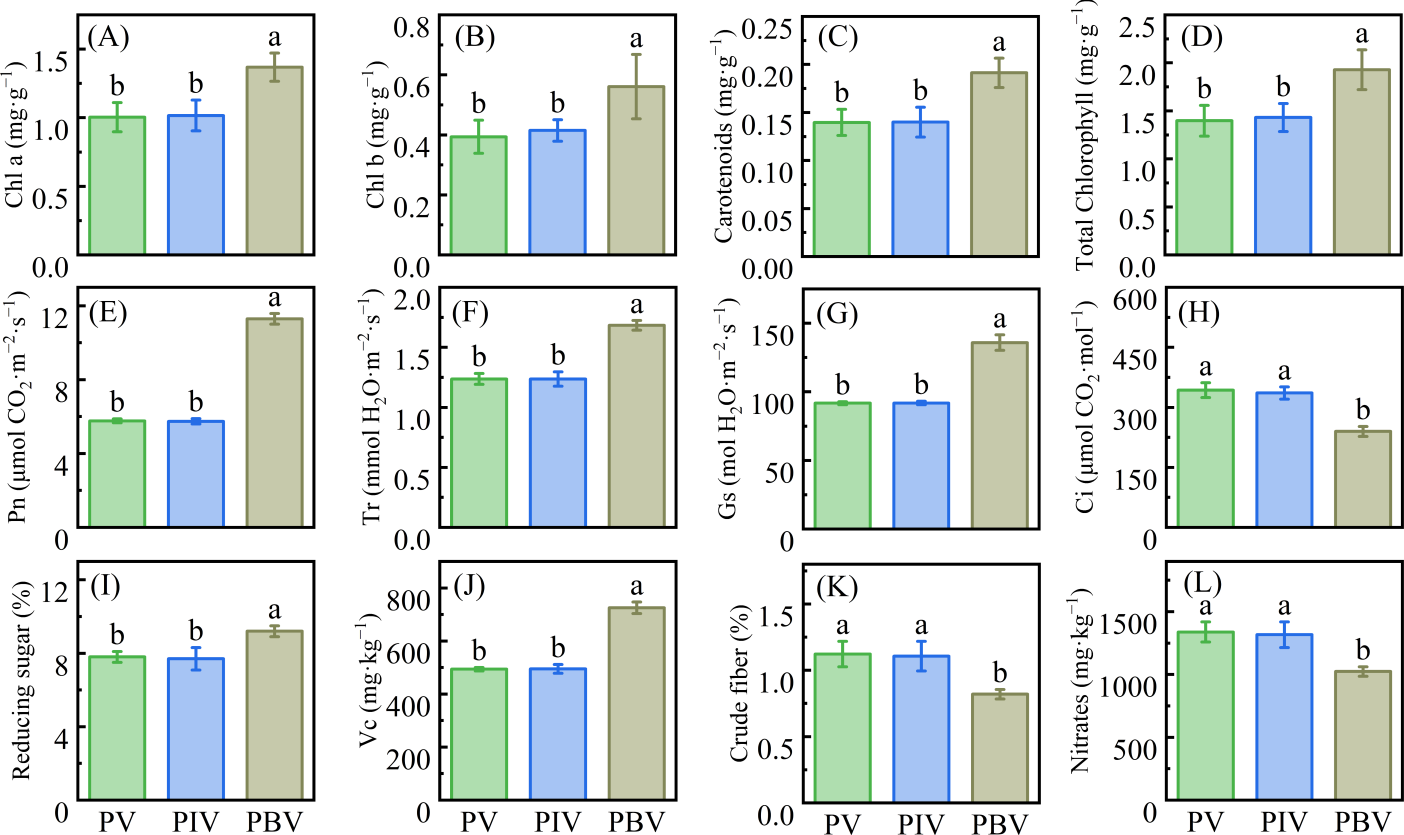
The effects of inoculating bacterial strains on Chl a (**A**), Chl b(B), carotenoids(C), total Chl (**D**), Pn (**E**), Tr (**F**), Gs (**G**), Ci (**H**), reducing sugars(I), Vc(J), crude fiber (**K**), and nitrates (**L**) of Chinese cabbage. Chl a: Chlorophyll a; Chl b: Chlorophyll a; Total Chl: Total Chlorophyll; Pn: Photosynthetic rate; Tr: Stomatal conductance; Gs: Intercellular CO_2_ concentration; Gi: Transpiration rate; Vc: Vitamin C. PV = phenol-contaminated soils + vegetable; PIV = phenol-contaminated soils + inactivated bacteria + vegetable; PBV = phenol-contaminated soils + active bacteria + vegetable. Different letters show significant differences (*P* < 0.05).

We found that the bacteria inoculation could bring a significant increase in the content of Chl a, Chl b, carotenoids, and total Chl in Chinese cabbage (*P* < 0.05; Fig. 4A through D). Furthermore, parameters related to photosynthesis, such as Pn, Tr, and Gs, were significantly increased, while Ci showed a significant decrease (*P* < 0.05; Fig. 4E through H). Compared to the PV treatment, it was found that the PBV treatment showed a significant increase in the content of reducing sugar and Vc by 17.95% and 46.93% (*P* < 0.05; Fig. 4I and J), respectively, while exhibiting a significant decrease in the content of crude fiber and nitrate by 27.00% and 23.52%, respectively (*P* < 0.05, Fig. 4K and L).

**Fig 5:**
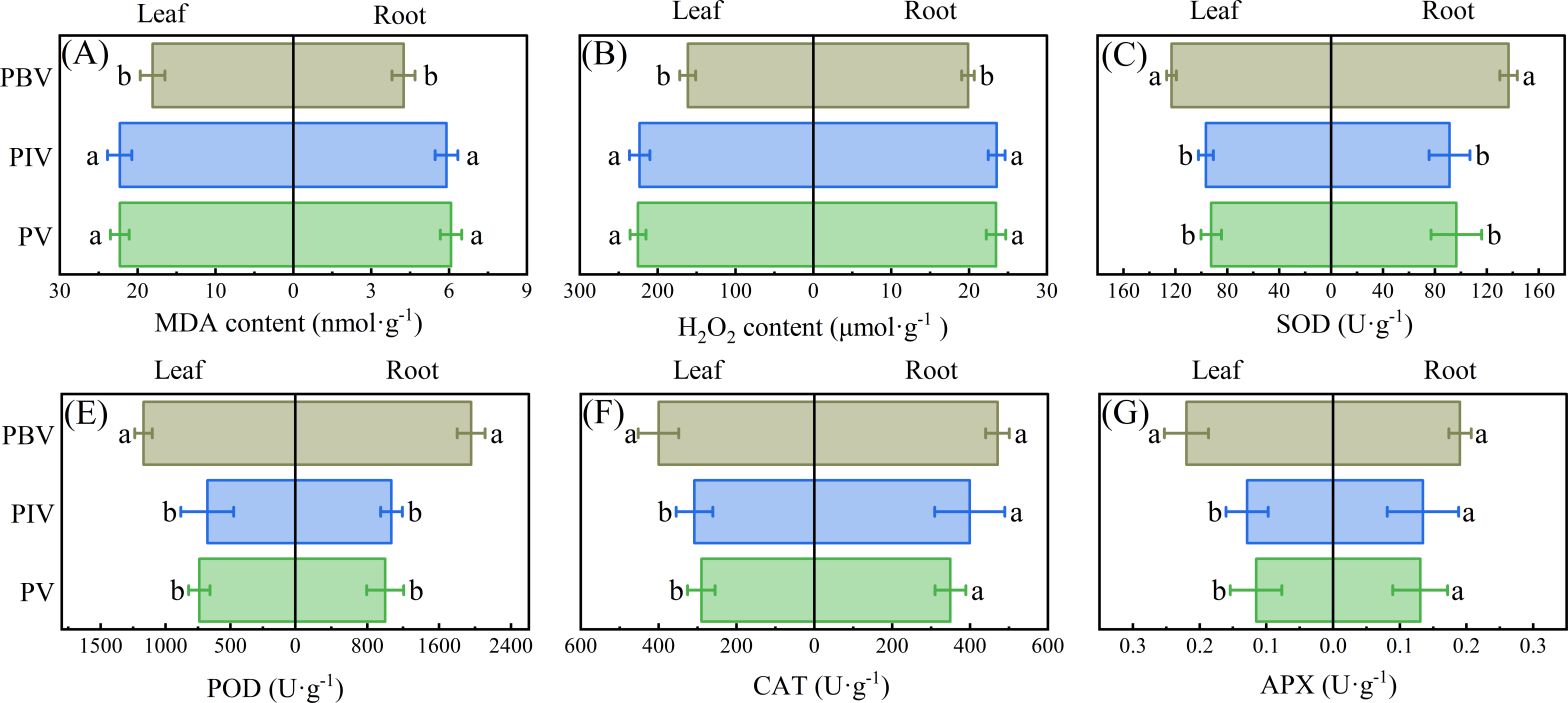
The effects of inoculating bacterial strains on MDA content (**A**), H_2_O_2_ content (**B**), SOD (**C**), POD (**D**), CAT (**E**) and APX (**A**) of Chinese cabbage. PV = phenol-contaminated soils + vegetable; PIV = phenol-contaminated soils + inactivated bacteria + vegetable; PBV = phenol-contaminated soils + active bacteria + vegetable. Leaf: Aboveground part of Chinese cabbage. Root: The underground part of Chinese cabbage. Different letters show significant differences (*P* < 0.05).

In addition, the inoculation with live bacteria reduced the levels of MDA and H_2_O_2_ in both the aboveground and underground parts of Chinese cabbage (*P* < 0.05; Fig. 5A and B). The activities of antioxidant enzymes, including SOD, POD, CAT, and APX, were significantly increased in both the aboveground and underground parts, showing an increase ranging from 33.07% to 95.32% (*P* < 0.05, Fig. 5C, E, F and G).

### Principal component analysis, heatmap analysis, correlation matrix, and PLS-PM analysis

**Fig 6:**
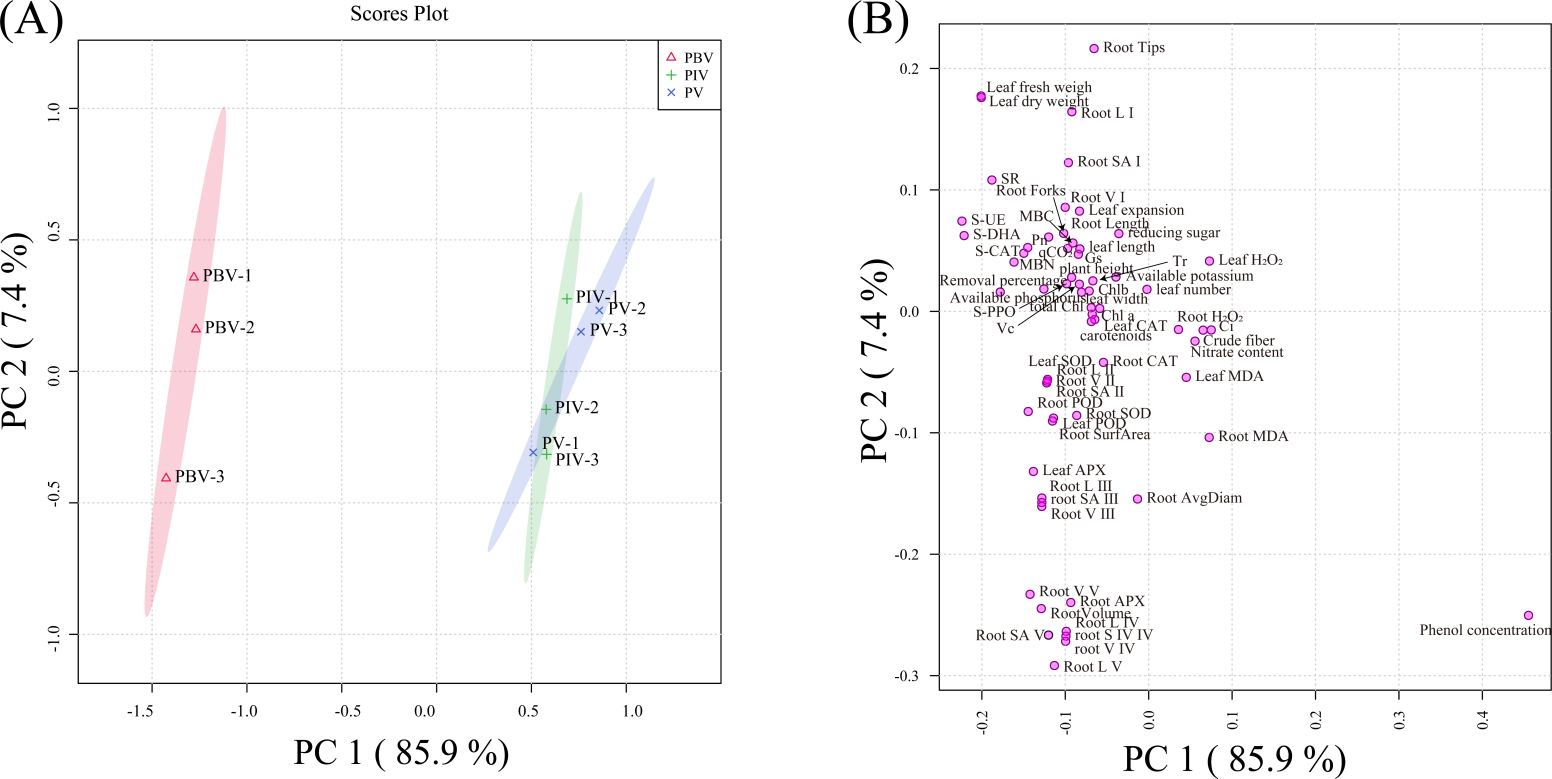
Variation in the aboveground and underground parts of the plants and various indicators of the soil among the treatments analyzed by PCA (**A**). Loading of the aboveground and underground parts of the plants and various indicators of the soil to first principal component (PC1) second principal component (PC2) (**B**).

**Fig 7:**
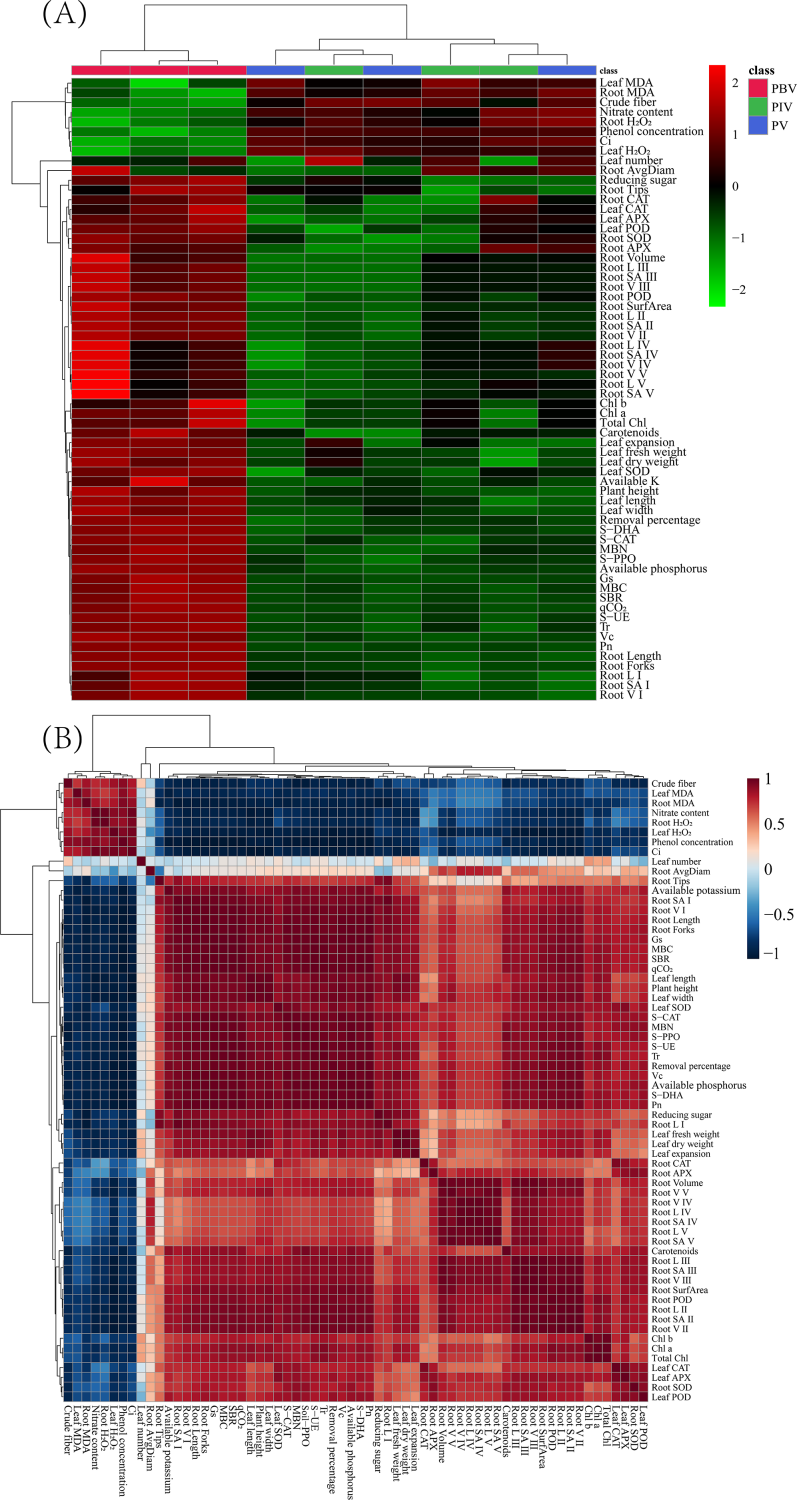
Heatmap analysis of various indicators (**A**). Correlation matrix showing correlation among the various indexes (**B**).

PCA was applied to depict the impact of inoculating bacterial strains on Potted Chinese cabbage. The indexes of soil and plant for the potted Chinese cabbage were well depicted by PC1 and PC2, which explained 85.9% and 7.4% of the total variation. PBV treatment showed significant separation from PV and PIV treatments (*P* < 0.05; Fig. 6A). Phenol concentration, Ci, and leaf H_2_O_2_ performed the most prominent positive influence on PC1, while S-UE, S-DHA, and leaf fresh weight had the greatest impact on PC1. Root tips, leaf fresh weight, and leaf dry weight brought the most substantial positive effect on PC2, whereas root SA IV, root V IV, and root L V showed the largest negative impact on PC2 (Fig. 6B). The soil phenol content showed a significant negative correlation with the activities of soil dehydrogenase, peroxidase, polyphenol oxidase, and urease (*P* < 0.05). Plant height, leaf length, leaf width, leaf fresh weight, leaf dry weight, root length, root surface area, and root volume were negatively correlated with phenol concentration, leaf MDA, root MDA, leaf H_2_O_2_, root H_2_O_2_, crude fiber, nitrate content, and Ci and positively correlated MBC, MBN, SBR, qCO_2_, S-DHA, S-CAT, S-PPO, S-UE, available phosphorus, available potassium, reducing sugar, Vc, Chl a, Chl b, carotenoids, total Chl, Pn, Tr, Gs, leaf SOD, leaf POD, leaf APX, and leaf CAT (Fig. 7).

**Fig 8:**
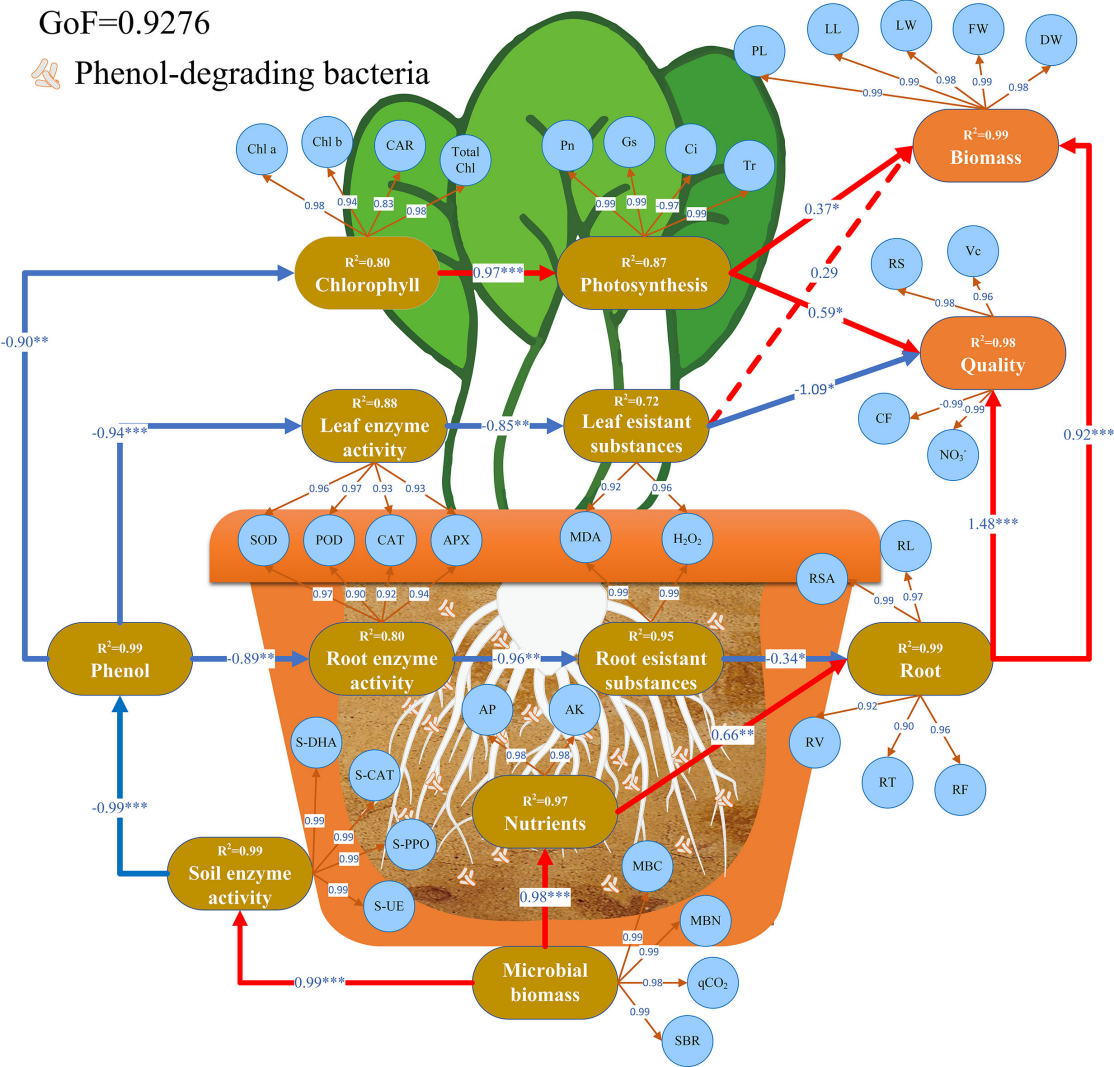
The relationship between soil microbial activity, soil enzyme activities, plant root system, plant antioxidant system, and plant biomass and quality by using PLS-PM under the conditions of inoculation with a phenol-degrading bacterium, *M. xuanwuensis* H13. The observed variables are represented by circles, and potential variables are represented by rounded rectangle. After 1,000 bootstraps, the path coefficients (between potential variables) and *R*^2^ (within ellipses) are calculated. Solid and dashed lines indicate significant (*P* < 0.05) and no significant (*P* > 0.05) correlations, respectively, and their colors represent the causal relationships that are positive (red) or negative (blue). The goodness of fit (GoF) statistical evaluation model is used to predict the overall performance.

In this work, a hypothetical model was established to quantitatively assess the relative contributions of various pathways associated with these factors (Fig. 8). Within the context of PLS-PM, the value associated with the arrow originating from latent results (depicted as rounded rectangles) to examined results (represented as circles) corresponds to the loading of the measured variable. The path coefficient (β) indicated on the arrow linking circles represents the overall effect of the latent variables. Positive values signify a positive impact on the outcome, while negative values indicate the opposite. The numerical value’s magnitude reflects the strength of the effect. Notably, the soil microbial biomass has a substantial influence on soil enzyme activity content (β = 0.99), as well as on the impact of soil enzyme activity on soil phenol content (β = −0.99). The introduction of H13 inoculation leads to a significant reduction (*P* < 0.05) in soil phenol content. Additionally, the soil microbial biomass exhibits a pronounced influence on soil nutrient content (β = 0.98), with nutrient levels exerting a greater effect on root systems (β = 0.66) in comparison to the impact of soil phenol on root systems via the subterranean antioxidant system (β = −0.34). Our model underscores that the growth and quality of Chinese cabbage are predominantly shaped by the intricate interplay of the photosynthetic apparatus, the antioxidant shield in aerial parts, and the subterranean root architecture. Within this framework, the root system emerges as the primary influencing factor, exerting a substantial impact on both the growth (β = 0.92) and quality (β = 1.84) of Chinese cabbage. While the influence of the aboveground antioxidant system on growth does not attain statistical significance (*P* > 0.05), it does exhibit a notable negative influence on the quality of Chinese cabbage (β = −1.09).

## DISCUSSION

In this study, a phenol-degrading bacteria *M. xuanwuensis* H13 was screened from a vegetable field that had been chronically contaminated with phenol-contained pesticides. The extensive use of pesticides brings worsening soil contamination by the intermediate compound phenol (69, 70). *M. xuanwuensis* H13 was isolated for the first time from soil samples in Jiangsu, China, which has been reported to be able to weather biotite (a silicate mineral) and release Si, Al, and Fe from the mineral (71). The ability of strain H13 to degrade phenolic pollutants was measured and reported in the current study. We observed a significant decrease in soil phenol content after H13 inoculation; moreover, H13 significantly increased phosphorus solubilization and potassium release, which increases the soil’s availability of these nutrients. Notably, H13 can promote the growth of Chinese cabbage on both aboveground and underground parts by being involved in the photosynthetic metabolism of plant roots.

Under the condition with increased volume of medium and reduced oxygen content, the phenol degradation capacity and growth ability of strain H13 performed decreased gradually due to its aerobic nature (72). The degradation of phenol by most phenol-degrading bacteria occurs under neutral conditions, and the availability of bacteria that can grow and maintain phenol degradation capability under extremely acidic conditions is limited (8). H13 showed a high degradation efficiency for 100 mg/L phenol within the external pH range of 5–9 after 24 h, with removal percentage reaching up to 80%. Furthermore, H13 maintained growth and strong ability of phenol degradation at pH 3 (removal percentage of 44%). *Rhodotorula* sp. ZM1 strain, which was screened from acidic mine drainage, showed the ability to degrade 1,100 mg/L of phenol in 120 h, achieving a removal percentage of 0.074 g/(g CDW h) cell dry weight at a pH of 3 (34). These findings indicate the feasibility of H13 to survive and remediate phenol contamination under extremely acidic conditions. In the presence of five different nitrogen sources, H13 showed a removal percentage of over 64% for phenol under sole carbon source with phenol, which suggests that H13 can degrade phenol under various environmental conditions (11, 73, 74).

The convergence of phytoremediation and microbial bioremediation strategies can build a more efficient approach to the remediation of contaminants, particularly organic compounds (23). Different effects were observed in the soil due to the application of phenol-degrading bacteria (PB), Chinese cabbage (PV), and the co-inoculation of Chinese cabbage with the degrading bacteria (PBV). The soil phenol content decreased by 40% in the Chinese cabbage cultivation (PV) compared with the control treatment (P). Cultivating Chinese cabbage alone to a certain extent can contribute to the reduction of phenol content in the soil. However, the phytotoxicity of pollutants may inhibit plant growth, reducing the efficiency of bioremediation (75). Using phenol-degrading bacteria (PB) and co-inoculation of Chinese cabbage with PBV reduced soil phenol content by 89% in the PB treatment and 93% in the PBV treatment. This result confirmed that H13 played a predominant role throughout the process of phenol degradation, which might be due to the even distribution of the degrading bacteria caused by the small soil volume in the potting environment (74).

S-DHA, S-CAT, S-PPO, and S-UE are important indicators of soil microbial activity, soil redox status, the degradation process of soil organic pollutants, and the stability and health of soil ecosystems (76–78). We found that the levels of S-DHA, S-CAT, S-PPO, and S-UE significantly increased after inoculation with H13 (*P* < 0.05). Simultaneously, there was a significant negative correlation between the enzyme levels and soil phenol content (*P* < 0.05; Fig. 6D). Xun et al. (79) found that the combined effect of rhizosphere growth-promoting bacteria with *arbuscular mycorrhizal* fungi and oats effectively remediated soil petroleum pollution. This approach led to a significant increase in the activities of urease, dehydrogenase, and sucrase in the soil. In the remediation study conducted by Raimondo et al. (80), the use of sugarcane filter cake to enhance the *Streptomyces* strains for the remediation of lindane-contaminated soil resulted in a significant increase in the activities of soil dehydrogenase, H_2_O_2_, and urease enzymes. These findings indicate that the inoculation of remediation microorganisms can enhance the activity of relevant enzymes in the soil, thereby promoting the degradation of organic pollutants (81). Furthermore, in the presence of both Chinese cabbage and the bacterial strains, the enzyme levels in the soil further increased, which could be closely related to the growth of plant roots in the soil and their interaction with plant growth-promoting bacteria (81, 82). The enhancement of root system and strain biological activities stimulates the secretion of hormones, enzymes, and other compounds (83). Our findings confirm that H13 works both independently and incorporate with plant root systems within phenol-contaminated soil. This interaction serves to enhance soil enzyme activity and concurrently reduce soil phenol content. However, it is worth noting that the degradation of phenol by a single plant species remains inefficient, and the associated potential toxicity of plants cannot be overlooked (84).

A high capacity for phosphorus and potassium solubilization can effectively increase the availability of nutrients in the soil, thereby promoting plant growth and enhancing plant resilience to stress (85). Our results showed that soils with H13 inoculation contained higher available phosphorus and potassium (77.74% and 20.19%). The increase in nutrient content in the soil promotes the absorption of nutrients by plant roots, thus enhancing plant growth (39, 86). We observed significant changes in the root system architecture of Chinese cabbage upon inoculation with H13, including an increase in the abundance of roots with a diameter ranging from 0 to 1.5 mm, as well as an increase in the number of root tips and forks (Table 2). These findings indicate that Chinese cabbage, under the conditions of inoculating H13, showed an enhanced development of root systems. The progression toward larger and more intricate root systems equips plants with an expanded surface area, fostering enhanced interactions with the soil.

Under the conditions of inoculating H13, the contents of SOD and POD in the aboveground tissues of Chinese cabbage exhibited a significant increase, as well as CAT and APX (*P* < 0.05). The significant increase in enzyme activity in this regard contributes to the removal of ROS within plant tissues (87, 88). We promisingly found that the levels of MDA and H_2_O_2_ in both the aboveground and underground parts of the plant showed significant reductions (*P* < 0.05; Fig. 5). Phenolic pollutants generate ROS that cause serious oxidative damage to the lipids and proteins of living cells and tissues (89). MDA and H_2_O_2_ are commonly used indicators for measuring the degree of oxidative stress (90, 91). Antioxidant enzymes play a pivotal role in converting excessive ROS and free radicals (commonly known as radicals) within the body into substances with reduced toxicity or harmlessness (92). This function helps maintain a balance in the levels of ROS within the SOD and dismutates O_2_•− to H_2_O_2_ and O_2_, while POD, CAT, and APX further catalyze H_2_O_2_ to H_2_O (93, 94). APX is also a key enzyme in the ascorbic acid-glutathione (AsA-GSH) cycle, contributing to maintaining intracellular redox balance by converting H_2_O_2_ to H_2_O (88, 95). H13 enhanced the levels of antioxidant enzymes in Chinese cabbage, facilitating the elimination of MDA and H_2_O_2_ within the plant. This process effectively curtails the harm inflicted by oxidative stress on the Chinese cabbage. Furthermore, the enhanced antioxidant capacity and stabilization of the photosynthetic system in the Chinese cabbage plants due to the presence of H13 bacteria had a direct impact on sugar accumulation. By minimizing oxidative stress and preserving the integrity of the photosynthetic apparatus, H13 allowed the plants to more efficiently convert light energy and carbon dioxide into carbohydrate molecules during photosynthesis. This increased photosynthetic productivity led to a greater buildup of sugars, such as sucrose, glucose, and fructose, within the Chinese cabbage tissues, which have implications for the quality and nutritional value of the Chinese cabbage produce.

*Bacillus marsiflavi* Bac 144 has been confirmed to enhance the activity of corn’s antioxidant enzymes in petroleum hydrocarbon-contaminated environment, eventually eliminating ROS in plants (83). The results obtained from the PLS-PM analysis revealed that substances resistant to root development (β = −0.34) appeared to have a relatively negative impact on the root system of Chinese cabbage compared to the positive effects caused by nutrient availability (β = 0.66). The elevated potassium content bolstered the plant’s resistance under unfavorable conditions, while the increased phosphorus content nurtured membrane lipids, effectively mending damaged membrane systems. These factors collectively fostered the expansion of the root system. Our results revealed a marked elevation in chlorophyll content and an augmentation in photosynthesis within Chinese cabbage under H13 inoculation. This phenomenon could be attributed to the reduction in ROS levels within the plant, thereby mitigating potential damage to chloroplasts (31, 96). The fortification of photosynthesis, the detoxification of ROS via antioxidant enzymes, and the growth of roots synergistically contribute to the upsurge in aboveground biomass and the enhancement of Chinese cabbage quality. Notably, the growth of root systems casts the most profound influence on aboveground biomass and Chinese cabbage quality. Moreover, the antioxidant system within the aboveground segment significantly impacts the overall quality (Fig. 7).

### Conclusions

The present study isolated *Myrides xuanwuensis* H13, a strain carrying phenol degradation capability. It showed environmental adaptability, and its ability to solubilize phosphorus and potassium proved effective in enhancing crop growth. In a potted experiment involving Chinese cabbage, the inoculation of *Myrides xuanwuensis* H13 facilitated the remediation of phenol pollution by elevating the activity of soil enzymes. Notably, the results obtained from our PLS-PM analysis confirmed that the primary effect of *Myrides xuanwuensis* H13 inoculation was the promotion of Chinese cabbage growth and quality through the stimulation of root development, while its impact on the photosynthetic system was comparatively secondary. The findings contribute to the development of sustainable agricultural practices, the understanding of microbial-plant interactions, and the potential application of microbial agents in environmental management. However, further characterization of the H13 strain, including its biosafety profile and detailed mechanisms of action, is needed before its widespread application can be recommended.

## Data Availability

The data are available from the corresponding author on reasonable request. The raw reads in this work were deposited on NCBI (https://submit.ncbi.nlm.nih.gov) with the accession number is OR024678.
